# Outlier Detection for Sensor Systems (ODSS): A MATLAB Macro for Evaluating Microphone Sensor Data Quality

**DOI:** 10.3390/s17102329

**Published:** 2017-10-13

**Authors:** Robert Vasta, Ian Crandell, Anthony Millican, Leanna House, Eric Smith

**Affiliations:** 1 Department of Mathematics, Virginia Tech, Blacksburg, VA 24061, USA; rvasta@vt.edu; 2 Department of Statistics, Virginia Tech, Blacksburg, VA 24061, USA; ian85@vt.edu (I.C.); lhouse@vt.edu (L.H.); 3 Department of Aerospace and Ocean Engineering, Virginia Tech, Blacksburg, VA 24061, USA; millican@vt.edu

**Keywords:** outliers, anomalies, acoustic arrays, analytics

## Abstract

Microphone sensor systems provide information that may be used for a variety of applications. Such systems generate large amounts of data. One concern is with microphone failure and unusual values that may be generated as part of the information collection process. This paper describes methods and a MATLAB graphical interface that provides rapid evaluation of microphone performance and identifies irregularities. The approach and interface are described. An application to a microphone array used in a wind tunnel is used to illustrate the methodology.

## 1. Introduction

The decline in cost and the increase in use of sensors has led to both an abundance of sensor data and the development of sensor data analytics (SDA) [1,2,3]. A critical problem in SDA is the evaluation of sensor quality, especially in a network of sensors. This article describes an analytical tool in MATLAB (Version R2016b, MathWorks, Natick, MA, USA) that is designed to quickly and effectively identify faulty sensors out of a set of possibly hundreds involved in an experiment, study or sensor system.

The original motivation for the development of our software draws from an issue naval engineers experience who perform acoustic tests with the aim of detecting undersea objects. Such acoustic tests are expensive, time-consuming, and rely on accurate, well-functioning microphones. However, verification of microphone data validity by specialized engineers would not take place for days, or sometimes weeks, after conducting tests. Inevitably, some verifications would indicate microphone failure, but, by the time of verification, re-testing would be impractical, if not impossible. This meant that when microphone errors were discovered, some data were deemed compromised and, potentially, unusable. Such large sensor systems are common to a number of settings including wind tunnel systems where microphones are used to estimate beamforms. As analysis of data from natural systems may have additional problems associated with identification of objects, we focus here on problems associated with microphone arrays in laboratory systems such as wind tunnels.

A variety of methods have been proposed for identifying outliers, anomalous observations and failing sensors. In a recent paper, for example, Liu and colleagues [4] developed an entropy based method to model the rate at which sensor readings degrade using data collected on a turbofan engine. Their first concern was to develop a method to select a subset of the sensors with which to train their model, which is described in [5]. This is done to reduce the computational burden of working with the entire data set. Liu et al. later extended this simple framework to a more complete one which incorporates correlation between individual sensors. As a measure of correlation, Liu et al. used the mutual information between two sensors. This quantity measures to what extent knowledge about one sensor reduces uncertainty about the other one. Due to its greater simplicity, our method does not require subsetting. Thus, we are able to consider the entire output from our sensor system.

Similar to the work of [4,5], Pang et al. [6] applied Gaussian process (GP) regression to anomaly detection in time series data. Pang’s approach can be described as a moving window based one-step-ahead prediction. Given an initial collection of data points, they train a GP model and then use it to predict the next input from the series. As their method is a one-step-ahead prediction based on a Gaussian model, it can be likened to a Kalman filter (see [7]). As with the work of Liu and colleagues, that of Pang et al. relies on a rather involved model to perform predictions. GPs scale poorly with data, which precludes their general application to datasets of the size we consider.

Hayes and Capretz [8] outlined a general approach to anomaly detection in big data settings. They were concerned both with quick and scalable detection, as well as taking good account of correlations between multiple sensors for more robust detection. Essentially, this is a univariate Gaussian predictor, using historical data to estimate a mean and variance. While simple, it is fast and lightweight, allowing monitoring of numerous sensors individually. Methods based on mean and variance may not be appropriate in cases where unusual observations significantly affect estimation of these quantities. The approach described below is quite similar in spirit to their method, however is nonparametric as the method uses interquantile ranges in place of standard deviations to measure discrepancy from the central signal.

Unlike any of the other work considered, our method is implemented in a lightweight and user friendly MATLAB interface. Our context also involves more user involvement with the data than those considered in the above papers [4,6,8]. While the previous papers consider industrial process monitoring applications, our data comes from a research application. Although this macro might be used for automated anomaly detection, our current focus is more on pointing out unexpected patterns in data for further inspection from researchers, and on identifying sensors that have failed.

## 2. Microphone Arrays

Modern wind tunnel testing involves multiple diverse sensor systems with anywhere from hundreds to thousands of individual sensors. These large-scale tests can be quite expensive and are usually time-sensitive. As such, any delays due to faulty instrumentation can have serious consequences. Equally serious is the possibility of discovering a sensor failure after the test has been completed because time and effort will have been spent collecting useless information. Although sometimes correctable, the time involved in doing so distracts the experimenter from achieving the experimental goals.

As such, any large scale sensor system needs methods to ensure that all the individual sensors are working as intended. Most commercial sensor systems contain rudimentary error detection for sensors within a given system, but these methods typically have no way of incorporating information about the ambient conditions under which they were run, or, more importantly, information from the output of other systems which are used in conjunction. This is a significant problem, as most large sensor systems in wind tunnel tests are made of smaller, unique sensor subsets. By combining the information from diverse sensor systems into a global error detection process, we can measure the extent to which sensors across systems are correlated and use that correlation to produce powerful predictions and error detection capabilities.

Thus, our work is motivated by two two questions: (1) Can we detect large-scale microphone issues during or immediately following tests? and (2) If a small percentage of microphones are malfunctioning, can we use the surrounding microphones to infer the true values of the bad microphones with a range and degree of certainty useful for analytics?

Question 1 focuses on prevention; if faulty microphones can be discovered on location, it is much less costly to replace the bad microphones and retest than if the bad microphones are detected at a later time. Question 2 focuses on salvaging imperfect data.

The analytical tool described in this paper aims to evaluate the first issue by automating much of the error identification process. The structure of the tool is described and evaluated using data from microphone arrays. Although the methods were developed specifically for microphone arrays, the program and tools can be useful in other situations where rapid assessment of sensor quality is required. We next discuss the statistical and graphical methods and their implementation. Results of a simulation are used to evaluate the error rate of the method for times series models. An example using data from a wind tunnel experiment is given to illustrate the package. Finally, some extensions are described. An appendix describes the macro in more detail and uses simulated data to illustrate the inputs and graphics.

## 3. Statistical Methods

Common failures with microphones include complete failure in which the microphone turns off resulting in a flat signal with low variability, drift of the signal and groups of signals that are different from regular signals. In addition, some microphones exhibit over-range and clipping. Over-range is defined here as having values that exceed normal ranges for the microphone. Clipping occurs when a value, usually at the upper-most or lower-most value, occurs multiple times.

The data set used in this analysis is viewed as consisting of measurements on K microphones or sensors. Each microphone is assumed to have the same starting and ending value and the same number of measurements. The measurements are indexed by a time sequence. Thus, we take $x_{ij}$ to be a measurement from microphone *i*, i=i,2,…,K at time *j*, j=1,2,…,T.

A standard approach for evaluating unusual observations in statistical analysis makes use of means and standard deviations of data at each time. However, this approach is limited because odd values can increase standard deviations and bias means resulting in reduced sensitivity. In addition, the approach requires a large number of sensors relative to the number of observations and the use may be limited due to correlations between measurements on the same sensor. For microphone data, it is not uncommon to have over 500,000 data values for a single microphone over a 30 s interval. Thus, we are typically dealing with a relatively small number of long series. In this work, the preference is to make use of a nonparametric approach that is less sensitive to extreme values and reduces correlation over time. Our approach is now described.

The first step involves segmenting the series, determining common behavior in each segment, and identifying outliers. The approach is to divide the series for a sensor into M segments of equal or roughly equal length. If L is the length of the segment, then M = T/L. It is convenient in many applications to take the length of the segment in proportion to the sampling rate as this will often result in all segments having the same length. We then re-index the data $x_{ij}$ for microphone *i* as $x_{ilm}$ with l=1,2,…,L and m=1,2,…,M. The second step is to define a statistic that summarizes the segment. For this purpose, we use a range that contains a specified percentage of observations, i.e., a range percentile. The range percentile is based on the quantiles from the data in each segment. Specifically, define the range percentile level α, that determines the upper and lower quantiles and hence the quantile range, IQRα. The value of IQRα is defined as the difference between the upper and lower quantiles, $Q_{p1}$ and $Q_{p2}$ within each segment where $Q_{p}$ is the value in the segment chosen so that p% of the values are less than $Q_{p}$ and p1−p2=α.

For example, for an α = 90% range percentile, use a quantile interval based the 5th and 95th quantiles of the $x_{ilm}$ for each microphone i and segment m. The IQR90 is the difference between the 5th and 95th quantiles. In our applications, the α = 90% quantile interval is used; however, in implementation, other values for α may be used. A larger value of α would result in identifying individual observations while a smaller value would focus on groups of observations. In addition, for applications, there are M segments per microphone, thus there are M interquantile ranges that we denote as $IQR\alpha_{im}$. With the range of typical data points provided by each $IQR\alpha_{im}$, outliers are determined based on the median and pseudo standard deviation (PSD) of the set of values (IQRs) for each microphone. Extreme values are defined as values that are more extreme than the median plus or minus a critical value times the PSD. When the number of segments is large, the critical value might be the appropriate quantile from the standard normal distribution (i.e., the critical *z* value). Other options might be to use a critical *t* value or adjusted *t* value as described below. The critical value is user defined and is associated with a desired level of certainty in correctly identifying sensor oddities.

In addition to identifying outliers, checks are also made to identify over-range data and clipped data. Over-range is determined by identifying observations that exceed specified limits of the microphone, while clipping is determined when an observed minimum or maximum observation duplicates, as if the data are censored for reasons that are unclear. For example, real data collected from accurate microphones often seem governed by a Gaussian distribution. Histograms of clipped data present large spikes in the tails in an otherwise Gaussian-shaped histogram and overranged data present two or more distributional modes. For this paper, we select candidate data for overranging and clipping by analyzing tail patterns and searching for histogram bins near the tails that are unusually large. Then, because data from the clipped microphones are more similar to Gaussian distributions than data from the overranged microphones, we run an Anderson–Darling test for normality [9,10] on samples from the microphones identified in the previous step, and we sort the microphones based on the results of this test.

The final check we make to assess data quality is to assess whether microphones, as a whole, are malfunctioning. Microphones that are not functioning may still emit a low signal, and thus may be determined to be off when a large number of observations are below some threshold that is user determined. Graphical displays are used to help distinguish different types of patterns and reduce false signals. These include power spectrum plots of microphones and histograms of observations for individual microphones. A heatmap is used to display the full set of oddities for comparison across microphones to identify patterns. The heatmap is a colored matrix that has M rows associated with segments and K columns associated with sensors. Four colors are used in the matrix, corresponding to a high extreme value, a low extreme value, a value that is off and a normal value.

## 4. Simulation Results and Sensitivity Measure

One critical decision in outlier and anomaly detection is the numerical criterion for deciding if an observation is sufficiently extreme to be deemed an outlier or potential outlier. While the actual determination might involve other factors and checks on the microphone, the determination of the cutoff is critical as too small a value will result in numerous false signals and too high a value in numerous missed outliers. To evaluate the method and estimate potential error rates, a simulation is used. Data were generated to mimic the microphone data in terms of the number of data points and segment size. Ten thousand series were generated from a variety of time series models (AR(1), AR(2) and ARMA) assuming normally distributed errors. Each series was of length 250,000 and segments of size 1000 were used, resulting in 250 evaluations. The statistical approach above was used to determine if a value was an outlier using two criteria: first, a cutoff of 3.0 PSDs (common in quality control methods) was used and, second, a cutoff was chosen based on the Bonferroni method. The cutoff of 3.0 is chosen from a normal distribution corresponding to an upper tail probability of approximately 0.0015; with the cutoff, the probability of observing a high outlier or more extreme would be 0.0015 (similarly for a lower tail outlier). While conservative for a single calculation, when there are many observations to assess, a fixed cutoff may result in a number of false signals. The Bonferroni method adjusts the cutoff so that the tail probability may account for the number of tests that are considered. Suppose the interest is in a γ overall error rate (e.g., 5%). Then, simply divide γ by the number of tests to specify the error rate for each test. The adjusted cutoff corresponds to the quantile from the normal or t-distribution associated with an upper tail probability of γ/(2T), where T is the number of tests.

The results of the simulation are summarized in Table 1. When γ=0.05 and a cutoff of 3.0 is used an excessive number of observations are flagged as odd observations when no correction is made. In the case of autoregressive models AR1, the error rate increases as the autocorrelation increases. A similar pattern holds for data generated from other time series models and error rates can be quite high. The Bonferroni method controls the error rate and results in a small rate of rejections as expected. As the autocorrelation increases, the Bonferroni rejection rate increases slightly, suggesting that the associated error rate may be affected by autocorrelation. Note that these results are for a situation with a large number of tests (250 in this case); when the number is smaller, the cutoff of 3.0 may be adequate. Thinning of the series may also be used to reduce correlation; however, this results in a loss of observations. Alternately, an adjustment to the sample size may be helpful (i.e., adjust degrees of freedom by multiplying by one minus the autocorrelation). In simulations, we found a sample size adjustment to result in fewer outliers although the approach tended to be conservative. Clearly, graphical displays and scientific judgement should be used to make final decisions about the quality of the observations and microphones. Simulated data are also used to illustrate the program and graphical displays in the appendix.

## 5. Evaluation of Microphone Array Data

**Figure 1:**
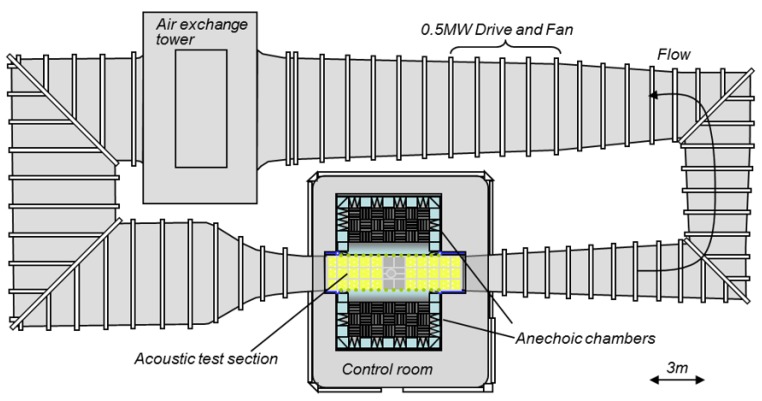
Virginia Tech wind tunnel where microphone array data were collected.

**Figure 2:**
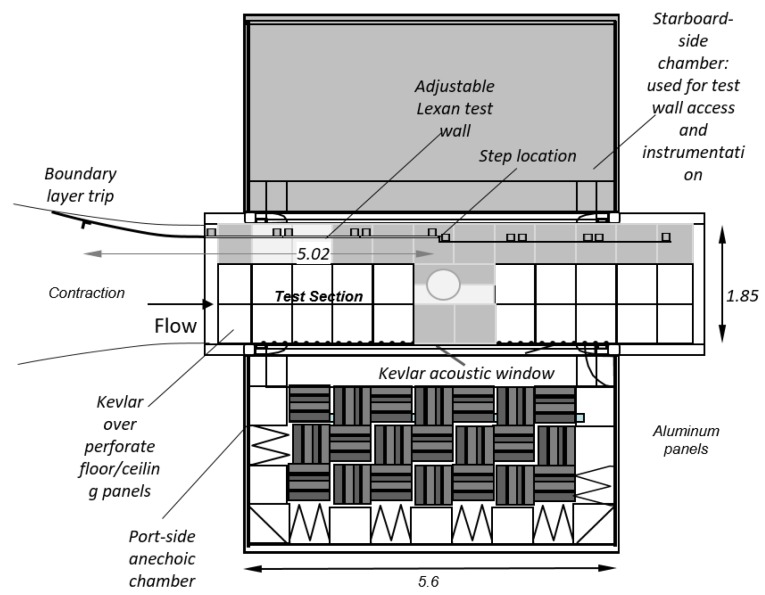
Top view of the test section used in the experiment to collect microphone array data, showing the anechoic chamber. All measurements are in meters.

**Figure 3:**
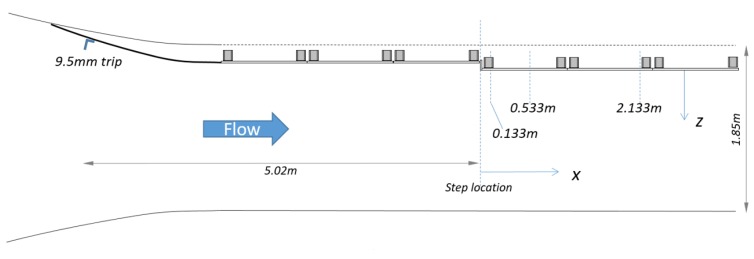
Top view of the experimental layout for collecting microphone array data.

This section describes an experiment that will be used as an example. The experiment is part of a study that focuses on the acoustic effects of a discontinuity or step in an otherwise smooth surface. For example, the surface of a ship may be mostly smooth except for discontinuities in the hull where plates are joined. Such steps may be acoustically loud and interest lies in the study of the effect of steps on acoustic characteristics of flow [11] and the design of efficient low-noise vehicles. The program was used to evaluate an experimental study using data collected in the Stability Wind Tunnel at Virginia Tech (Blacksburg, VA, USA) (Figure 1). The wind tunnel allows for experiments with anechoic (free from echo) or semi-anechoic conditions. The data evaluated below are part of a study on pressure fluctuations produced by forward steps immersed in a turbulent boundary layer [11]. Figure 2 shows a schematic of the test section, test wall and anechoic chambers. In a typical study, sidewalls are constructed of Kevlar cloth; however, for this study, one wall was removed and replaced with an adjustable wall that is suitable for growing high-Reynolds number two-dimensional turbulent boundary layers (Figure 3).

**Figure 4:**
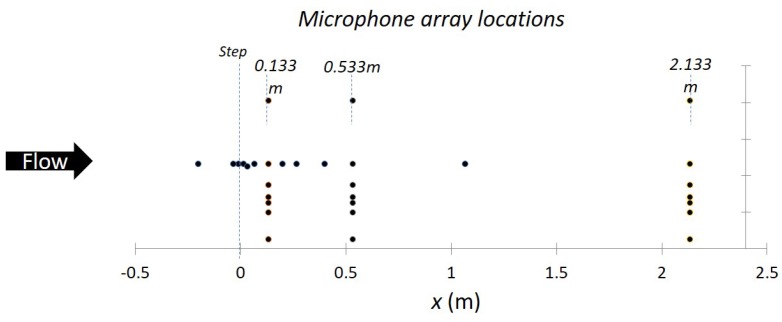
Locations of microphones to collect array data.

In the overall experiment, step sizes of 3.6, 14.6 and 58.3 mm are used with flow speeds of 30 and 60 m/s. A variety of measurements are made including the mean wall Cp, mean velocity profiles, 3D turbulence profiles and wall pressure spectra. Below, we evaluate one part of the experiment with a focus on wall pressure spectra. Measurements are made using 31 wall-mounted microphones placed forward and aft of a sharp step at a particular location (Figure 4). A sampling rate of 51,200 Hz is used. To segment the data, segments of size 51,200 are used resulting in 20 segments. A cutoff of *z* = 3.02 is used for the critical value, based on the Bonferroni method adjusting for 20 tests.

**Figure 5:**
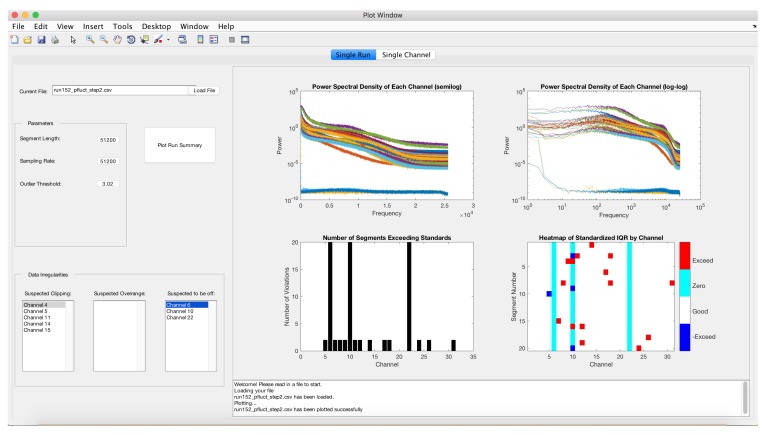
Spectral densities and plots identifying unusual segments in the microphone array data.

**Figure 6:**
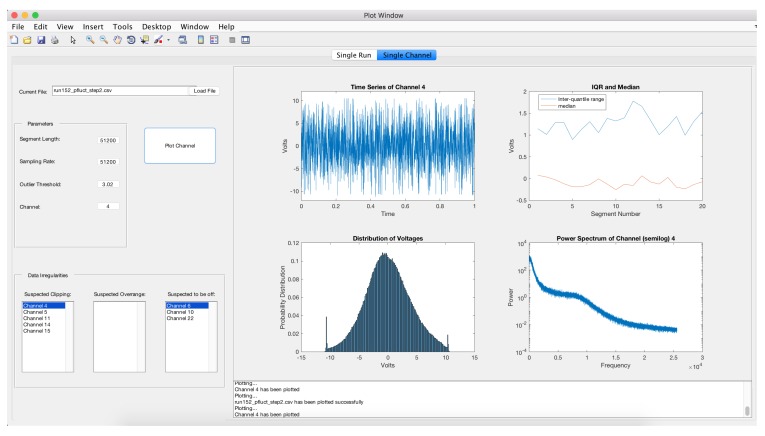
Graphical displays for channel 4 in the microphone array data.

**Figure 7:**
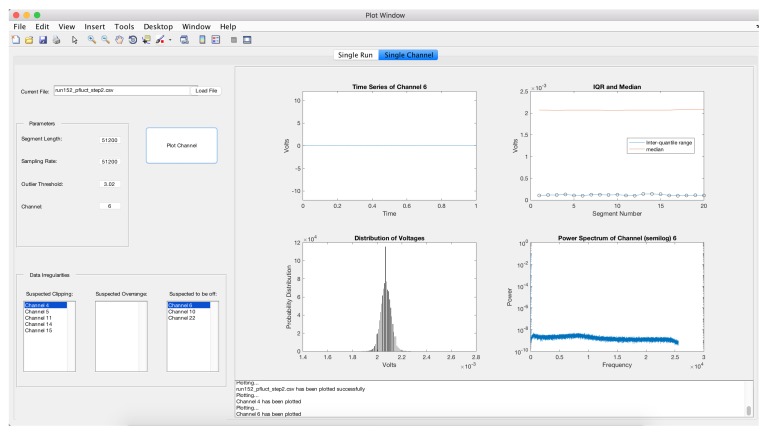
Graphical displays for channel 6 in the microphone array data.

Diagnostic plots of the 31 channels are presented in Figure 5 and suggest problems with several microphones. Three microphones are identified as being off and five channels suggest clipping. The three channels have power spectra that are considerably different from the other microphones. This is also illustrated in the segment bar chart and the heatmap. A variety of other channels have minor violations. A plot of channel 4 illustrates clipping (Figure 6). Note that the histogram plotted in the lower left plot shows peaks in the distribution at the upper and lower values. Channel 6 is one channel that is identified as being off and this is indicated by the flat time series, the narrow histogram and simple power spectra (Figure 7).

## 6. Conclusions

The MATLAB Graphical User Inteface (GUI) allows rapid evaluation of microphone data quality and identifies extreme segments, microphones that are not functioning as well as clipping and overrange. In an example with 31 microphones, we identified three microphones that were not functioning and several microphones with clipping. Identification of these issues may lead to improved analysis of the information in the microphone system. The GUI provides some flexibility for a user. This includes selection of the critical threshold for identifying unusual groups of observations. It is recommended that the user use the GUI with some data of known quality to identify the threshold prior to use on experimental data. We have found the threshold of three to be somewhat liberal with real data, i.e., it tends to identify unusual observations that do not affect resulting analyses. Evaluation of individual series using a Bonferroni correction can also be helpful in selecting the critical threshold.

The GUI can be extended in a number of ways. First, more interactive plots can be added. Although not included in this version of the GUI, we have a feature that allows interactive evaluation of data by allowing the user to click on a suspected spectrum to visualize the series that is suspect. A future implementation will focus on systems with multiple sets of sensors and correlated systems. The current analysis treats the microphones as providing independent information. To incorporate correlation, a Gaussian Process approach is being developed that allows for both correlation between microphones of the same and different types of sensors while maintaining the nonparametric nature of the analysis.

## Figures and Tables

**Table 1:** Percentage of observations determined to be acceptable or rejected as outliers using a cutoff of 3.0 or the Bonferroni cutoff for various time series models. The model parameters are given in parentheses. A false rate of rejection of γ = 0.05 (5%) is expected for the Bonferroni method.

2*Model	Cutoff = 3		Bonferroni Cutoff	
	% Accept	% Reject	% Accept	% Reject
AR1(0.0)	63	37	95	5
AR1(0.5)	61	39	96	4
AR1(0.8)	56	44	94	6
AR1(0.9)	52	48	91	9
AR1(0.95)	43	57	88	12
AR2(0.5,0.2)	59	41	94	6
AR2(0.8,0.1)	51	49	92	8
AR2(0.6,0.2)	56	44	93	7
AR2(0.95, −0.1)	55	45	93	7
AR2(1.2,−0.3)	56	44	94	6
ARMA(0.9,0.5)	52	48	92	8
ARMA(0.5, 0.5)	62	38	95	5
ARMA(0.995,−0.1)	15	85	86	14

**Figure A1:**
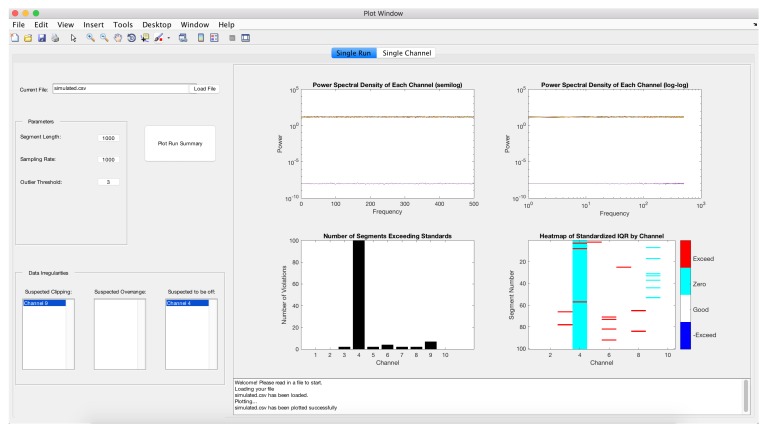
Power spectra and graphical displays for identifying unusual segments in the simulated data set.

**Figure A2:**
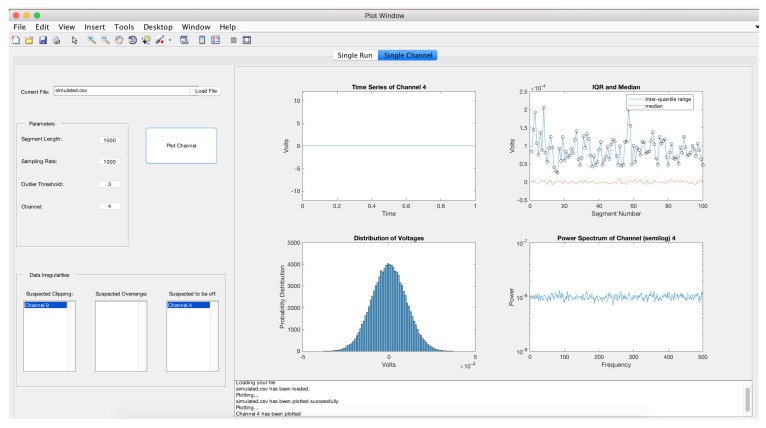
Graphical displays for channel 4 of the simulated data set.

**Figure A3:**
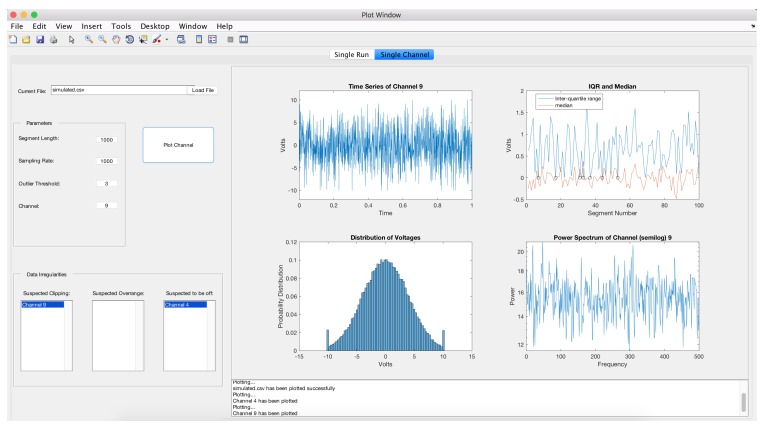
Graphical displays for channel 9 of the simulated data set.

## Appendix A. Visuals and Graphical User Interface

In this appendix, the Matlab graphical user interface (GUI) and associated displays are described. The interface has two sections or tabs. Analytical methods described earlier are used with the first tab to identify channels and segments that are suspect. The second tab is designed to display information about selected channels. Information to be used in the analysis is input into the left-hand side of the GUI. The data set to be used in the analysis is a .csvfile and its location is entered as part of the first tab. The .csv file needs to be organized beforehand so that each column is a time series of a specific microphone, e.g., channel. Additional data input includes the segment length, sampling rate and the measure of extreme sensitivity. The segment length is the number of time points that is used to determine a natural window for evaluating the interquantile range. For example, if a measurement series consists of 100,000 points, one might divide the series into M = 100 segments with segment length of size L = 1000. The number of channels corresponds to the number of columns in the input file and is populated when the file is read. The sampling rate is the number of points per unit of time. The input associated with extreme sensitivity is the cutoff for evaluating points that are extreme; it is set at z = 3 as a default. From here, we have two tabs associated with the application. The single run tab will generate plots associated with channels while the single channel run provides plots to evaluate specific channels. Once a tab is selected and all inputs are made, selecting the “Plot Run” box will generate the set of graphical displays. Each set is further described.

### Appendix A.1. Single Run Tab

The single run tab provides visual displays of data from every microphone channel (column in the data set). We use four plots to visualize information from every microphone. The top left plot displays a view of the power spectral densities of each microphone, and the top right plot displays the same information on a logarithmic scale. These plots are useful for identifying patterns in the power spectra that may be different than the others. The two lower plots display potential errors in the data collection. Each run is segmented into a number of smaller parts or segments. For a given α, we calculate the IQRα and the median for each segment. Then, we check for segments that are extreme i.e., are more extreme than the median IQRα plus or minus z times the pseudo standard deviation of the IQRα, and for segments that are consistently within 0.001 of 0. The GUI provides plots on the bottom left for the number of segments flagged for each microphone, and a plot on the bottom right displays a heatmap of which segments are flagged, with a color code used to indicate why they have been flagged. A box at the bottom of the figures summarizes the analyses that have been run and will indicate if any input errors have been occurred.

The second main feature of the single run tab is that, as the plots are being generated, it is also highlighting potentially bad microphones and sorting them into three categories: suspected clipping, suspected overrange, and suspected to be off. To populate the ‘off’ bin, we use the information from the heatmap calculations and find microphones with multiple segments identified as off. For clipping and overrange, we create a histogram for each microphone. We found that while data from accurate microphones generally may be described by a normal distribution, clipped microphones have large spikes in the tails and overranged microphones have bimodal distributions. We pick out candidates for overranging and clipping by analysing tail trends and searching for histogram bins near the tails that are unusually large. Then, because the clipped microphones exhibit a bit more normal behavior than the overranged microphones, we run an Anderson–Darling test for normality [9,10] on samples from the microphones identified in the previous step, and we sort the microphones based off the results of this test. Channels that are suspected to have irregularities are identified by number and reported in the lower left box of the display. Boxes at the bottom left identify channels that have been flagged and reasons for the flag. Plots are generated using the single channel tab by clicking on the tab and inputing the channel to be investigated.

### Appendix A.2. Single Channel Tab

When the single channel tab is selected, visualizations for a single microphone channel data are produced and may be used to verify the data inconsistencies identified in the run tab. After a channel is selected, four plots are produced. The top left plot shows the time series data for the indicated channel. The top right plot shows the α = 90th percentile IQR${}_{\alpha }$ and median for each segment. Circles are used to indicate segments that have a positive statistic that exceeds the critical value. The bottom left plot is a histogram of the time series data. This can be used to quickly inspect and verify the potential errors highlighted in the single run tab. Lastly, in the bottom right corner, there is a plot of the power spectral density.

## Appendix B. Simulated Data Example

To illustrate the use of the application, we use a simulated data set using 10 channels of data. In addition, 100,000 data points were generated for each channel. Most of these channels are described as sine waves with a peak at 1000 HZ, adding normally distributed random noise with mean 0 and standard deviation 4 to the values. Channel 4, however, has a standard deviation of .001, so it should be flagged as a microphone that is off, and Channel 9 has had all values greater than 10 reduced to 10, and similarly all values less than −10 have been increased to −10. This channel should be flagged for clipping. A segment length of 1000 is used, thus the macro tool divides each series into 100 segments.

We start by loading in our sample dataset, simulated.csv. We click on the Single Run tab and input the needed parameters, and then we click the Plot Run Summary button to generate plots and find data irregularities. Figure A1 shows the resulting screen. We see in the data irregularities section that Channel 9 has been flagged for clipping and Channel 4 has been flagged for being off; we will investigate these channels further in the Single Run Tab. However, first, we will consider the four plots generated by the Plot Run Summary button.

The top two plots are the power spectral density of each channel. The top left plot has an *x*-axis on semilog scale, whereas the top right plot uses a log–log scale. Power spectral density plots show how the power of the signal is distributed across various frequencies; a peak at a certain frequency indicates an underlying periodic signal. Looking at the power spectrum for this dataset, we see a nearly horizontal line, which is indicative of normally distributed simulated data. Note that there are two lines, one associated with the majority of the channels and the other associated with an anomalous channel.

The bottom two plots are two ways of visualizing anomalies in each record for each channel. For each channel, the IQRα of each record is computed. The pseudo standard deviation of these IQRαs is computed for each channel, and any records with an IQRα that is sufficiently far from the median of the IQRαs are flagged. A high IQRα relative to the other records can be indicative of an abnormally large measurement, which increases the likelihood of clipping. In addition, any records with IQRα within a certain range of zero ($10^{-3}$ is used) are flagged as being essentially zero. The bottom right plot is a heatmap that displays the violations. The heatmap is essentially a matrix of the IQRαs with a color assigned to the relative magnitude; blue indicates an unflagged or within compliance record, cyan is a record that is zero, and red is a record flagged for having a large IQRα and white suggests a record that is considered in compliance. The bottom left plot is a bar chart that displays the number of records that were flagged for each channel. In the case of channel 4, all records were flagged; for other channels, only a few records exceeded the threshold. Note that, with a normal distribution, the probability of exceeding three standard deviations is 0.0027 so less than one observation is expected, on average, to exceed 3.

If it is decided to investigate a channel further, click on the Single Channel tab. An additional entry box is added and the channel number is a new input parameter to specify the channel to plot. After inputting Channel 4 and pressing the Plot Channel button, we get the plots shown in Figure A2. The first plot is a time series of the data in a segment. The second plot is a graph of the IQR and median for each of the segments. In this case, there are 100 segments and the median is essentially zero (note the y-axis is multipled by $10^{-4})$. This plot is interactive; a circle on the plot of the IQR indicates a segment that is high. By clicking on a circle (or any join point of the graph), the associated time series of the segment is graphed in the left plot. The bottom left plot is a histogram of the data and the bottom right plot displays the power spectrum.

Another example of an anomaly for this data is channel 9. If this plot is selected, then a different set of plots occurs in Figure A3. The top left plot shows the time series data for Channel 9. The top right plot shows the median and IQRα for each record; this is the data that is associated with one column of the heatmap from the Single Run tab. Circles indicate a record that has been flagged. In the bottom right, we have the power spectral density for this channel. In the bottom left plot, we have a histogram of the time series data. We typically expect to see a normal distribution here, and, for Channel 4, the histogram does look normal. However, Channel 9, which was flagged for clipping, produces a plot with peaks in the tails of the histogram where the values greater than ten were clipped to 10 and values less than −10 were set to −10 (Figure A3).
